# Delirium in the ICU: an overview

**DOI:** 10.1186/2110-5820-2-49

**Published:** 2012-12-27

**Authors:** Rodrigo Cavallazzi, Mohamed Saad, Paul E Marik

**Affiliations:** 1 Division of Pulmonary, Critical Care, Sleep Disorders University of Louisville, Louisville, KY, USA; 2 Division of Pulmonary and Critical Care, Eastern Virginia Medical School, Norfolk, VA, USA; 3 Department of Medicine, Eastern Virginia Medical School, 825 Fairfax Avenue, Suite 410, Norfolk, VA, 23507, USA

**Keywords:** Delirium, Critical illness, Coma, Sedatives, Antipsychotics

## Abstract

Delirium is characterized by a disturbance of consciousness with accompanying change in cognition. Delirium typically manifests as a constellation of symptoms with an acute onset and a fluctuating course. Delirium is extremely common in the intensive care unit (ICU) especially amongst mechanically ventilated patients. Three subtypes have been recognized: hyperactive, hypoactive, and mixed. Delirium is frequently undiagnosed unless specific diagnostic instruments are used. The CAM-ICU is the most widely studied and validated diagnostic instrument. However, the accuracy of this tool may be less than ideal without adequate training of the providers applying it. The presence of delirium has important prognostic implications; in mechanically ventilated patients it is associated with a 2.5-fold increase in short-term mortality and a 3.2-fold increase in 6-month mortality. Nonpharmacological approaches, such as physical and occupational therapy, decrease the duration of delirium and should be encouraged. Pharmacological treatment for delirium traditionally includes haloperidol; however, more data for haloperidol are needed given the paucity of placebo-controlled trials testing its efficacy to treat delirium in the ICU. Second-generation antipsychotics have emerged as an alternative for the treatment of delirium, and they may have a better safety profile. Dexmedetomidine may prove to be a valuable adjunctive agent for patients with delirium in the ICU.

## Definition

Delirium is a syndrome of several different etiologies characterized by a disturbance of consciousness with accompanying change in cognition. Characteristic features of the syndrome include impaired short-term memory, impaired attention, disorientation, development over a short period of time, and a fluctuating course [1]. Not all described features need to be present for the diagnosis of delirium, and the intensity of the symptoms ranges widely among patients. One of several approaches to classify delirium is to divide it into motoric subtypes. Three subtypes of delirium are recognized based on the pattern of symptoms: hyperactive, hypoactive, and mixed [2]. Physiologically, delirium is characterized by a derangement of cerebral metabolism with cerebral dysfunction and is usually caused by a general medical illness, intoxication, or substance withdrawal [1,3]. The syndrome of delirium encompasses a few distinct entities with unique pathophysiology and clinical manifestations. These include sepsis-associated encephalopathy, alcohol withdrawal syndrome, and hepatic encephalopathy.

## Epidemiology

In a multicenter study, the prevalence of delirium in ICU patients was 32.3% [4]. In specialized ICUs, the prevalence of delirium may be higher. For instance, a study showed a prevalence of delirium as high as 77% in ventilated burn patients [5]. The incidence of delirium in the ICU ranges from 45% to 87% [6-8]. The incidence appears to vary according to whether the studied population is composed exclusively of mechanically ventilated patients. As an example, a study found an incidence of delirium of only 20% in nonintubated ICU patients [9], whereas another study found an incidence of 83% in mechanically ventilated patients [10].

The two most common types of delirium in the ICU are mixed and hypoactive [11]. Hypoactive delirium tends to occur more frequently in older patients compared with other types of delirium and has a worse prognosis. In a study of patients who underwent elective surgery with postoperative ICU admission, the 6-month mortality was 32% in patients with hypoactive delirium compared with 8.7% in those with other types of delirium [12].

## Pathophysiology

**Figure 1:**
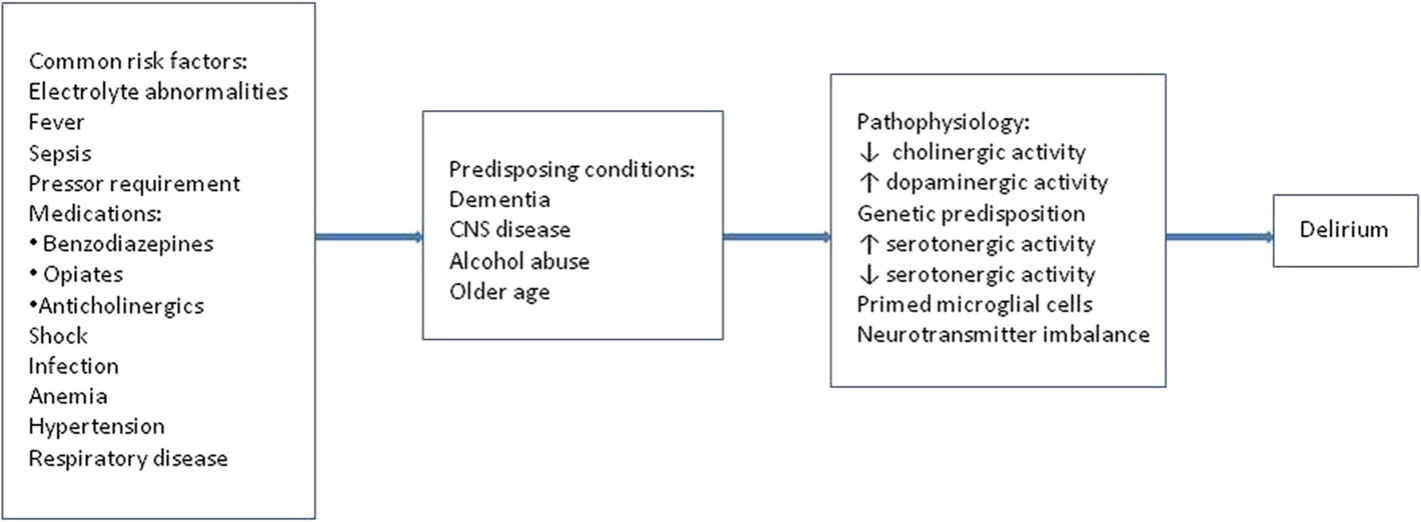
Factors leading to delirium.

Different mechanisms have been proposed to explain the pathophysiology of delirium. However, these mechanisms are not mutually exclusive and it is likely that they often act in concert (Figure 1). One hypothesis postulates that decreased cholinergic activity may lead to delirium [13]. This hypothesis is supported by the observation that anticholinergic medication use is associated with increase in delirium symptoms [14] and that patients with delirium have higher serum anticholinergic activity compared with those without delirium [15].

Acetylcholine down regulates inflammation. Thus, it is not surprising that there is an imbalance between inflammatory and anti-inflammatory mediators in delirium, with increased levels of inflammatory mediators and a blunted anti-inflammatory response [16]. In this light, the role of inflammation and its consequent deranged coagulation has been explored in a recent cohort study of mechanically ventilated ICU patients. In this study, five markers of inflammation and four markers of coagulation were measured in the plasma of patients. After adjustment for potential confounders, including severity of illness, higher plasma concentrations of the inflammatory marker soluble tumor necrosis factor receptor-1, and lower plasma concentrations of the coagulation marker protein C were associated with increased risk of delirium. However, an unexpected finding was that lower plasma concentrations of matrix metalloproteinase-9, another inflammatory marker, were associated with higher risk of delirium [17]. Another mechanism implicated in the pathophysiology of delirium is overactivity of the dopaminergic system. Clinically, evidence for this comes from case reports associating bupropion, an antidepressant with dopamine and norepinephrine activity, with development of delirium [18]. Furthermore, a genetic basis for increased dopaminergic system-induced delirium has been substantiated by the demonstration that mutant genes leading to lower cerebral dopamine activity are protective against delirium [19].

Both increased serotonergic activity and a relative serotonin deficiency also have been associated with delirium [20]. A high serotonergic state in association with delirium has been classically described in patients with the serotonin syndrome, a condition often emerging from the interaction of medications leading to increased serotonergic effects and that in its most severe form presents with hyperthermia, muscle rigidity, and multiple organ failure [21]. On the other hand, low levels of tryptophan—an amino acid that crosses the blood brain barrier and is a precursor to neurotransmitters serotonin and melatonin—have been associated with delirium after surgery in patients older 50 years [22]. Another study found that either high or very low levels of tryptophan are independently associated with an increased risk of delirium in ICU mechanically ventilated patients [23]. Whereas decreased serotonin activity may be implicated in the development of delirium, it also is possible that the production of other metabolites of tryptophan, such as kynurenine, leads to pathway activity that results in neurotoxins predisposing to delirium [24].

Patients who are more prone to delirium, such as the elderly or those with underlying central nervous system disease, also may have heightened central nervous system response to inflammatory mediators. It appears that these patients may have an increased number of microglial cells, which are primed and can be readily activated in response to a mild stressor [25].

The amino-acid neurotransmitter system has a prominent role in the pathophysiology of alcohol withdrawal syndrome. In particular, chronic alcohol exposure may lead to a decrease in the number of and function of gamma aminobutyric acid receptors and an increase in the *N*-methyl-D-aspartate receptors. Both mechanisms could predispose patients to alcohol withdrawal syndrome [26,27].

## Clinical manifestations

Delirium typically manifests as a constellation of symptoms with an acute onset and a fluctuating course. These symptoms have been organized into cognitive and behavioral groups. Common cognitive symptoms include disorientation, inability to sustain attention, impaired short-term memory, impaired visuospatial ability, reduced level of consciousness, and perseveration. Common behavioral symptoms include sleep-wake cycle disturbance, irritability, hallucinations, and delusions [28]. The manifestations of delirium can vary widely among patients. Whereas some patients may manifest somnolence and even coma, others appear anxious, disruptive, or combative [29]. Recognition of this symptom variability has led to the classification of delirium into motoric subtypes. One such subtype is hyperactive delirium, of which the manifestations include agitation, hypervigilance, irritability, lack of concentration, and perseveration. Hypoactive delirium manifests as diminished alertness, absence of or slowed speech, hypokinesia, and lethargy. Mixed delirium, as the name implies, includes manifestations of both hyperactive and hypoactive delirium [2].

The clinical manifestations also vary according to the precipitating factors. For instance, patients with bacteremia often present with encephalopathy and declined mental status [30]. Conversely, patients with alcohol withdrawal syndrome present with symptoms of an overactive sympathetic central nervous system [31]. As a consequence, patients with alcohol withdrawal syndrome commonly have agitation, insomnia, tremor, tachycardia, and hypertension [32].

## Assessment of delirium

A number of instruments are available to detect delirium in critically ill patients. The importance of using these instruments lies in that most cases of delirium in the ICU go undetected. Indeed, there is evidence that even when prompted to report delirium, ICU physicians recognize less than one third of delirious critically ill patients when they are not using an instrument to aid in their diagnosis [33]. In a systematic review from 2007, six validated instruments to assess delirium in critically ill patients were identified. These included the Cognitive Test for Delirium, abbreviated Cognitive Test for Delirium, Confusion Assessment Method for the Intensive Care Unit (CAM-ICU), Intensive Care Delirium Screening Checklist, Neelon and Champagne Confusion Scale, and the Delirium Detection Score [34]. Another instrument to detect delirium is the Nursing Delirium Screening Scale, of which the validity and reliability were assessed in the ICU [35]. Table 1 summarizes these diagnostic instruments [8,36-40].

**Table 1:** Instruments for the diagnosis of delirium in the ICU

**Instrument**	**Assessment features**	**Assessment method**	**Diagnosis**
Abbreviated Cognitive Test for delirium [36]	Total score obtained by summing up two content scores: attention (range 0–14) and memory (range 0–10)	Memory is assessed by recognition of pictured objects. Attention is assessed using the visual memory span subtest of the Wechsler Memory Scale-Revised.	<11
Confusion Assessment Method for the ICU [8]	The instrument assesses four features: 1) acute onset of mental status changes or fluctuating course; 2) inattention; 3) disorganized thinking; 4) altered level of consciousness	Feature 1: assess for acute change in mental status, fluctuating behavior or serial Glasgow Coma Score or sedation ratings over 24 hours. Feature 2: assess using picture recognition or random letter test. Feature 3: assess by asking the patient to hold up a certain number of fingers. Feature 4: rate level of consciousness from alert to coma.	Features 1 or 2 are positive, along with either Feature 2 or Feature 4
Intensive Care Delirium Screening Checklist [37]	Checklist of eight items: altered level of consciousness, inattention, disorientation, hallucination or delusion, psychomotor agitation or retardation, inappropriate mood or speech, sleep/wake cycle disturbance, and symptom fluctuation. The presence of each item of the scale is attributed one point.	The scale is completed based on information collected from the entire shift. Items scored in a structured way with definitions available for every item.	≥4
Neelon and Champagne Confusion Scale [38]	The scale is divided into three subscales: 1) information processing (attention, processing and orientation); 2) behavior (appearance, motor and verbal behavior); and 3) physiological condition (vital function, oxygen saturation, and urinary incontinence). The subscales contain a total of nine items. The score ranges from 0 through 30. Each item is scored according to the severity of the symptom.	Information based on observations by nurses at bedside. Items scored in a structured way with definitions available for every item.	Moderate to severe delirium (0–19); mild to early delirium (20–24); at high risk for delirium (25–26); no delirium (27–30)
Delirium Detection Score [39]	Eight criteria: agitation, anxiety, hallucination, orientation, seizures, tremor, paroxysmal sweating, and altered sleep-wake rhythm. Each criterion has four severity levels and accounts for 0, 1, 4, or 7 points depending on severity of the symptom.	Assessment performed during each shift by the treating physician and nurse who used a form with the items and definitions. The highest score in each shift was recorded. Items scored in a structured way with definitions available for every item.	>7
Nursing Delirium Screening Scale [40]	This scale contains five items: disorientation (verbal or behavioral manifestation of not being oriented to time or place or misperceiving persons in the environment); inappropriate behavior (behavior inappropriate to place and/or for the person, such as pulling at tubes or dressings, attempting to get out of bed when that is contraindicated, and the like); inappropriate communication (communication inappropriate to place and/or for the person, such as incoherence, noncommunicativeness, nonsensical or unintelligible speech); illusions/hallucinations (seeing or hearing things that are not there or distortions of visual objects); and psychomotor retardation (delayed responsiveness or few or no spontaneous actions/words). Symptoms are rated from 0 to 2 based on the presence and intensity of each symptom. Total score is obtained from the addition of the symptom ratings. Maximal score is 10.	Assessment performed per shift by bedside nurses.	>1

The most extensively studied instrument is the CAM-ICU, which was validated to assess delirium at the bedside in nonverbal ventilated ICU patients [41]. Using a structured format, this tool evaluates four features, namely, acute onset or fluctuating course, inattention, disorganized thinking, and altered level of consciousness. When administered by bedside nurses with no formal psychiatric training, the CAM-ICU demonstrated high accuracy (sensitivity of 93% to 100% and specificity of 98% to 100%) and interrater reliability (K = 0.96) in a single-center study [10]. In another study, the CAM-ICU was systematically applied by bedside nurses in the ICU during an implementation process that involved training of the nurses. The agreement between the assessment from bedside nurses and a research staff rater was low at baseline but very high during the implementation process [42]. However, subsequent studies have shown that the CAM-ICU has a more modest sensitivity ranging from 64% to 81%, whereas the specificity remains high ranging from 88% to 98% [33,43,44]. In a more recent study, CAM-ICU had a high specificity (98%) but a rather low sensitivity (47%) [45]. The contrast between the latter study and others [42,46] may stem from different implementation processes, that is, different approaches to training and education of providers applying the tool.

Two studies have compared different instruments for detection of delirium in critically ill patients [33,43]. In one study, CAM-ICU was prospectively compared with the Intensive Care Delirium Screening Checklist in 126 patients. CAM-ICU showed superior sensitivity (64% vs. 43%) but lower specificity (88% vs. 95%) [33]. In another study, the accuracy of three instruments (CAM-ICU, Nursing Delirium Screening Scale, and Delirium Detection Score) was compared in a prospective study of 156 patients. Although the sensitivities of CAM-ICU and the Nursing Delirium Screening Scale were similar (81% for CAM-ICU; 83% for Nursing Delirium Screening Scale), the CAM-ICU showed superior specificity (96% vs. 81%). The Delirium Detection Score showed a sensitivity of 30% and a specificity of 91% [43].

The above-mentioned instruments are our best tools for the early detection of delirium in the ICU, but their widespread application has some limitations. First, studies show quite different sensitivities for the same instrument, particularly the CAM-ICU. The difference in sensitivities may be explained by heterogeneity in the patient populations included in the studies but more notably by differential level of training and experience among the assessors in the studies. Thus, it is difficult to establish how accurate these instruments are without adequate training, but it is reasonable to infer that a substantial proportion of critically ill patients with delirium will remain undiagnosed if these instruments are applied by inexperienced or nontrained health care providers. In support of this notion, two recent systematic reviews pooled several studies evaluating the accuracy of CAM-ICU [47,48]. The majority of the studies included in the systematic reviews showed that the CAM-ICU is a highly accurate instrument for the diagnosis of delirium in the ICU. However, in the only study that was performed in a nonresearch setting, most patients with delirium were not detected by CAM-ICU [45,47].

Whether these instruments can be feasibly implemented in busy nonacademic ICUs is an important issue. Furthermore, it is not well established that the systematic application of these instruments influences the outcomes of critically ill patients. However, there is evidence that when delirium screening is applied as part of a broader protocol initiative that includes active management of sedatives and analgesics as well as nonpharmacological measures, such as music and reassurance, several clinical benefits may ensue, such as shorter duration of mechanical ventilation, lower ICU and hospital stay, and lower 30-day mortality [49]. The protocol also is associated with cost savings [50].

## Biomarkers

Several biomarkers have been associated with delirium. Serum anticholinergic activity is enhanced in patients with delirium, and the number of symptoms of delirium increases with higher serum anticholinergic activity level [15]. The S100B protein is an indicator of glial activation and/or death; thus, it is a nonspecific marker of brain injury [51] The S100B protein has been shown to be elevated in patients with delirium [52]. Recently, emphasis has been given to the study of inflammatory biomarkers for the prediction of delirium. For instance, McGrane et al. evaluated 87 critically ill patients in a study; the majority of them had sepsis upon admission to the ICU. They found that higher baseline levels of procalcitonin or C-reactive protein were associated with more days with delirium [53]. Other investigators have found that the profile of increased inflammatory biomarkers changes in critically ill patients with delirium according to the presence or absence of clinical evidence of inflammation (infection or systemic inflammatory response syndrome) [54]. Additional serum biomarkers shown to be elevated in patients with delirium include brain-derived neurotrophic factor, neuron-specific enolase, interleukins, and cortisol [55,56]. Whereas the use of biomarkers for delirium is promising, because they can provide diagnostic and prognostic information, more validation studies are necessary before they can be applied in clinical practice.

## Risk factors for delirium

In a study of non-ICU patients who underwent hip fracture repair, older age and male sex have been associated with an increased and independent risk of delirium [57]. A systematic review that included six observational studies evaluated risk factors for delirium by multivariate analysis. Twenty-five risk factors were significantly associated with delirium, and among those four were recognized as predisposing to delirium: respiratory disease, older age, alcohol abuse, and dementia. Twenty-one risk factors were considered precipitating, because they were related the patient's underlying disease; some of these included electrolyte abnormalities, fever, pressor requirement, increasing opiate dose, and metabolic acidosis [58]. Medications are an important risk factor for delirium, especially in the elderly. Classes of medications commonly associated with delirium include anticholinergic agents, benzodiazepines, and opiates [59]. In the ICU, benzodiazepines appear to have a more prominent role in the development of delirium [60].

## Prognosis

Ely *et al.* evaluated the effect of delirium on 6-month mortality and length of hospital stay among 224 critically patients receiving mechanical ventilation in a prospective cohort study. Delirium was assessed daily by study nurses with the use of CAM-ICU. After adjusting for clinically relevant variables, including age, severity of illness, comorbid conditions, and use of sedatives and analgesic medications, delirium remained associated with a 3.2-fold increase in 6-month mortality and a 2-fold increase in hospital stay duration [61]. Outcomes of critically ill patients are influenced not only by the presence of delirium but also the duration of it. In a multicenter study, 354 mechanically ventilated patients had daily assessment for delirium with the use of CAM-ICU. After adjustment for age, severity of disease and other covariates, delirium was associated with a 2.5-fold increase in short-term mortality, and there was a dose-response increase in mortality with increasing duration of delirium. Patients who had delirium for 1 day had 14.5% all-cause 30-day mortality, whereas the figure was 39% for those with 3 days or more of delirium [62]. In another cohort study, 304 patients admitted to a single ICU were evaluated daily with use of CAM-ICU. After adjustment for age, severity of illness, and other covariates, every additional day of delirium in the ICU was associated with a 10% increase in the hazard of death within 1 year post ICU admission [63]. Delirium in the ICU also is associated with more mechanical ventilation days, longer ICU stay, and longer hospital stay [64]. In patients whose symptoms do not fulfill criteria for a formal diagnosis of delirium, the presence of psychomotor agitation—an individual manifestation of delirium—is associated with increased risk for death after adjustment for Acute Physiology and Chronic Health Evaluation Score (APACHE), age, and the presence of coma [65].

In addition to leading to an increase in hospital stay and mortality, delirium is associated with long-term cognitive impairment. For instance, in a cohort study of 77 patients who underwent mechanical ventilation, more than 70% of them had cognitive impairment at 1 year follow-up. Increasing duration of delirium was independently associated with cognitive impairment after adjustment for several covariates, including education and preexisting cognitive function [66]. In another cohort study of 1,292 ICU survivors, quality of life questionnaires were sent to patients 18 months after ICU discharge. The study had an overall response rate of 71%. Although there was no statistically significant difference in quality of life between patients with delirium and those without delirium, more pronounced cognitive failure as determined by self-reported cognitive failure questionnaire was found in patients with delirium after adjustment for covariates [67].

## Nonpharmacological therapy

Nonpharmacological therapies have an important role in both the prevention and treatment of delirium. As an example, a study in 852 elderly patients admitted to a hospital showed that an intervention strategy against delirium led to a 40% decrease in the odds of developing delirium. The strategy comprised protocols that targeted risk factors for delirium, such as dehydration, immobility, sleep deprivation, visual impairment, cognitive impairment, and hearing impairment [68]. Although this study was performed in non-ICU patients, it is reasonable to infer that components of the intervention also are effective in critically ill patients. In this light, other authors have emphasized the importance of environmental factors in the risk of developing delirium in the ICU, and some strategies have been proposed to mitigate the impact of delirium. These include noise reduction, natural light exposure at daytime, minimization of artificial light exposure at nighttime, ambient temperature optimization, and improved communication [69].

**Figure 2:**
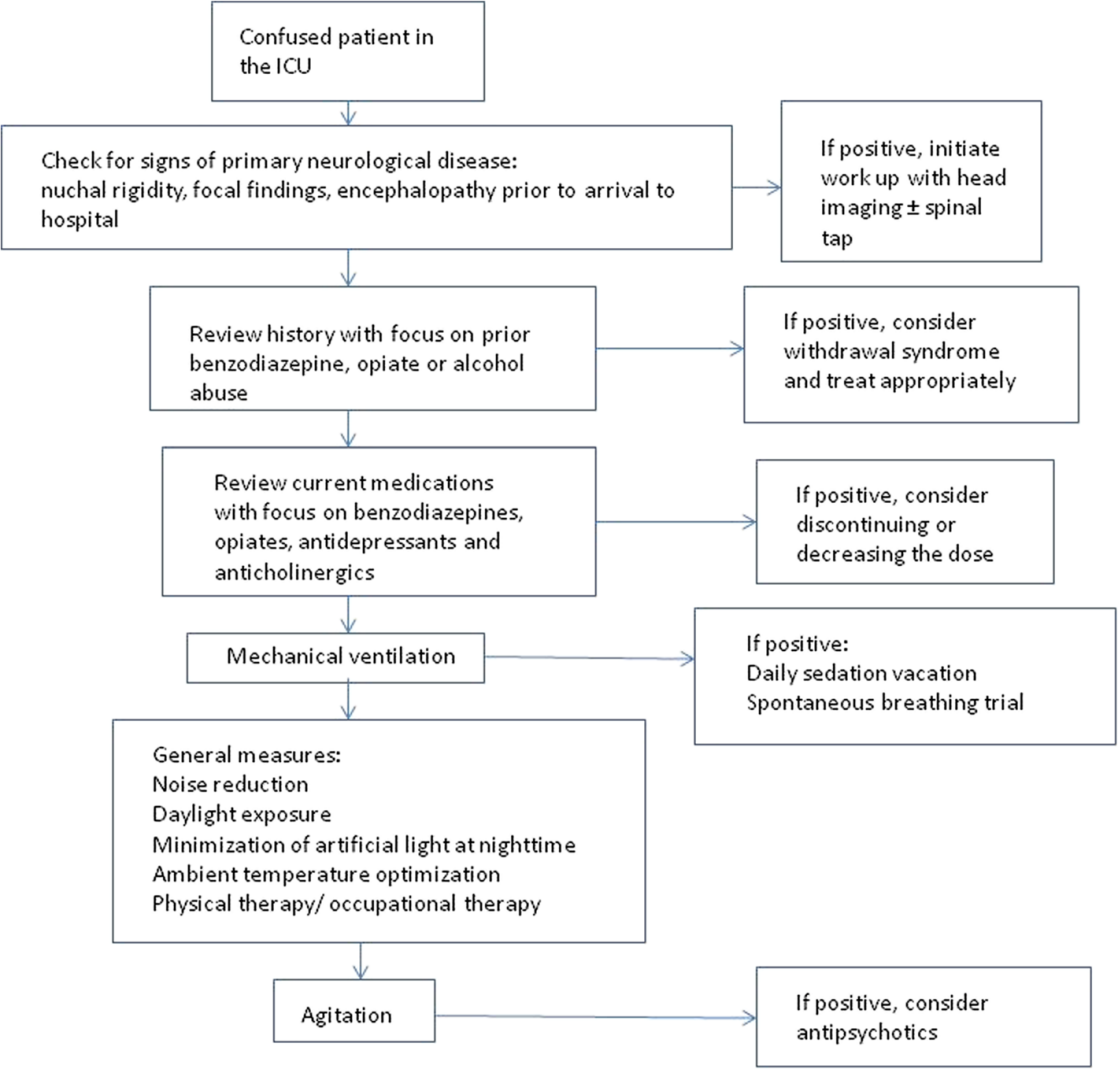
Proposed strategy for the initial management of patients with delirium in the ICU.

Noise in the ICU is known to disturb patients’ sleep [70]. Furthermore, it has been suggested that a disturbed sleep may influence the risk of delirium. The impact of noise on the quality of sleep and thus on the risk of delirium has been illustrated in a recent clinical trial that demonstrated that the use of earplugs at nighttime leads to better sleep and less confusion [71]. Limiting the exposure to sedatives also may have beneficial effects on the risk of delirium. A randomized, clinical trial showed that protocolized daily interruption of sedatives associated with spontaneous breathing trials leads to significantly shorter duration of coma in mechanically ventilated patients but no significant change in delirium in the assessable patients [72]. The addition of physical and occupational therapy to daily interruption of sedation leads to shorter duration of delirium and better functional status in mechanically ventilated patients [73]. Figure 2 presents a proposed strategy for the initial management of patients with delirium in the ICU.

## Pharmacological therapy

### Sedatives

Sedatives have the potential to promote delirium [74]. In an observational study, lorazepam was an independent and statistically significant risk factor for development of delirium whereas other sedatives, such as propofol and opiates, had no statistically significant association with delirium [60]. In a randomized, double-blind trial, 30 hospitalized AIDS patients with delirium were assigned to treatment with haloperidol, chlorpromazine, or lorazepam. Treatment with haloperidol or chlorpromazine resulted in significant improvement in the symptoms of delirium and low prevalence of extrapyramidal side effects. Patients treated with lorazepam had no improvement in delirium and developed treatment-limiting adverse events [75]. Thus, benzodiazepines are generally avoided for the treatment of delirium in hospitalized patients. In fact, because benzodiazepines are an important risk factor for delirium in critically ill patients, limiting their use may decrease the overall incidence of delirium in the ICU. It should be noted, however, that in patients with alcohol withdrawal syndrome, benzodiazepines are the recommended therapy [76]. Furthermore, benzodiazepines should not be abruptly discontinued in patients with benzodiazepine dependence [27].

Dexmedetomidine is a highly selective α2-adrenergic receptor agonist that provides analgesia and “cooperative sedation” without important effects on respiratory status [77,78]. It may be a suitable sedative agent for mechanically ventilated patients with delirium or agitation in whom extubation is being considered, a group for which there is little data. A meta-analysis of clinical trials that included nonelective critically ill patients or patients after high-risk elective surgery showed that dexmedetomidine led to a modest reduction in length of ICU stay (−0.48 days; 95% CI −0.18 to −0.78 days; *P* = 0.002) but no significant difference in delirium, mortality, and length of hospital stay. The review was weighed on by studies that included patients who underwent high-risk elective surgery. In addition, the meta-analysis was limited by significant heterogeneity among the included studies, but one important finding was that the use of both a loading dose and a high maintenance dose of dexmedetomidine led to a significantly increased risk of bradycardia (5.8% vs. 0.4%; *P* = 0.007) [78].

Dexmedetomidine appears to be particularly effective to decrease the risk of delirium compared with benzodiazepines in mechanically ventilated ICU patients. Compared with lorazepam, dexmedetomidine led to a statistically significant increase in days alive without delirium or coma (median 7 vs. 3; *P* = 0.1) in a randomized, controlled trial of 106 patients [79]. More recently, Jakob et al. published the results of two clinical trials; one compared dexmedetomidine with midazolam and the other dexmedetomidine with propofol. Although there was no change in length of ICU and hospital stay, those who received dexmedetomidine were more able to arouse, cooperate, and communicate their pain. Dexmedetomidine also led to a reduction in duration of mechanical ventilation compared with midazolam but not compared with propofol. Importantly, dexmedetomidine led to more bradycardia and hypotension compared with midazolam and more first-degree atrioventricular block compared with propofol [80]. Furthermore, there have been reports of patients receiving dexmedetomidine who developed bradycardia and subsequently pulseless electrical activity [81,82]. Thus, caution should be exercised in the elderly, patients with underlying heart disease, and those who develop bradycardia while receiving dexmedetomidine.

### Antipsychotics

The first-generation antipsychotic haloperidol has been used traditionally for treatment of delirium. Indeed, the 2002 clinical practice guidelines on sedatives recommend haloperidol as the agent of choice for the treatment of delirium [74]. There also is evidence that haloperidol may be beneficial in preventing delirium in a select group of ICU patients [83]. Patients taking haloperidol should have electrocardiographic monitoring for QT interval prolongation and arrhythmias. In the critical care setting, haloperidol is usually given as an intermittent intravenous injection [74]. More recently, there have been studies that evaluated the efficacy of second-generation (atypical) antipsychotic medications in ICU patients (Table 2) [84-87].

**Table 2:** Clinical trials evaluating antipsychotics in critically ill patients with delirium.

**Author, year**	**No. of patients**	**Inclusion criteria**	**Interventions**	**Blinding**	**Randomization**	**Primary endpoint**	**Results**
Reade [84], 2009	20	Mechanical ventilation, inability to extubate because of agitation	Dexmedetomidine 0.2-0.7 mcg/kg/h (loading dose was optional) Haloperidol 0.5-2 mg/h (loading dose was optional)	No	Computer-generated random sequence	Time from commencement of study drug to extubation	Patients on dexmedetomidine were extubated sooner than those on haloperidol: 9.9 (IQR 7.3-24) vs. 42.5 (IQR 23.2-117.8) hours, *P* = 0.016.
Girard [85], 2010	101	Mechanical ventilation, abnormal level of consciousness, receipt of sedative or analgesic medications	Haloperidol 5 mg Ziprasidone 40 mg placebo. Second dose administered 12 hours after the first if QT < 500 msec; then every 6 hours.	Yes	Computer-generated, permuted-block randomization scheme	Number of days alive without delirium or coma	No significant difference in number of days alive without delirium or coma. *P* = 0.66. Haloperidol: 14 (IQR 6–18) days Ziprasidone: 15 (IQR 9.1-18) days Placebo: 12.5 (IQR 1.2-17.2) days
Devlin [86], 2010	36	ICU patients with delirium and an order for as-needed haloperidol	Quetiapine 50 mg every 12 hours titrated upwards on a daily basis if haloperidol was needed. Placebo.	Yes	Computer-generated random sequence	Time to first resolution of delirium	Time to first resolution was shorter with Quetiapine therapy than with placebo, *P* = 0.001. Quetiapine: 1 (IQR 0.5-3) days Placebo: 4.5 (IQR 2–7) days
Skrobik [87], 2004	73	ICU patients with delirium	Haloperidol 2.5-5 mg every 8 hours Olanzapine 5 mg daily	Only those assessing outcomes	Even/odds day basis	Not specified	No difference in delirium index scores, *P* = 0.83. No difference in benzodiazepine use, *P* = 0.9.

IQR, interquartile range.

## Haloperidol for prevention of delirium in the ICU

In a randomized, double-blind trial from two centers, the effect on delirium prevention of intravenous haloperidol (0.5 mg followed by an infusion at 0.1 mg/h over 12 hours) was compared with placebo in 457 patients older than 65 years who were admitted to the ICU after noncardiac surgery. Haloperidol led to a significant decrease in the incidence of delirium within the first 7 days after surgery (15.3% vs. 23.2%; *P* = 0.031) and a decrease in length of ICU stay (21.3 h vs. 23 h; *P* = 0.024). Although haloperidol was associated with lower 28-day mortality, this was not statistically significant (0.9% vs. 2.6%; *P* = 0.175) [83]. That the patients included in this study were not so ill (as determined by their mean APACHE II score < 9) is a potential drawback of this study. Another limitation is the absence of an outcome determining the patients’ functionality, such as ability to return to independent living [88].

## Comparison of haloperidol with second-generation (atypical) antipsychotic medications

In a clinical trial that included 73 ICU patients, oral haloperidol was compared with olanzapine for the treatment of delirium. There was no difference in reduction in delirium severity between the groups; however, 13% of the patients who received haloperidol developed mild extrapyramidal symptoms, whereas none of the patients in the olanzapine group had these side effects. The study design was limited by inadequate randomization method, small sample size, and lack of blinding from the treating physician and nurses. In addition, the study had no placebo group [87].

A clinical trial, including 101 patients on mechanical ventilation with abnormal level of consciousness, found no difference in number of days alive without delirium or coma in patients treated with haloperidol, ziprasidone, or placebo. There was no statistically significant difference in extrapyramidal symptoms among the three groups of patients. Limitations of this study included a small sample size and the large proportion of patients (42%) in the placebo group who received open-label haloperidol [85].

## Comparison of haloperidol with dexmedetomidine

A randomized, open-label trial compared haloperidol with dexmedetomidine in 20 patients with agitated delirium in the ICU. The ICU length of stay was significantly decreased by 5 days in those who received dexmedetomidine. Limitations of this study included lack of blinding and the small sample size [84].

## Comparison of second-generation (atypical) antipsychotic medications with placebo

A randomized, double-blind trial compared quetiapine with placebo in 36 critically ill patients with delirium. All patients were allowed to receive intravenous haloperidol. The time to resolution of delirium was significantly shorter with quetiapine therapy than with placebo; the decrease was by 3.5 days (*P* = 0.001). This study was limited by small sample size, performance of multiple statistical analyses (which increases the odds of type 1 error), and the low enrollment rate, which is the result of stringent inclusion criteria [86].

## Final considerations on the use of antipsychotics for treating and preventing delirium in the ICU

In summary, the evidence for use of antipsychotics for treating delirium in the ICU is weak. The studies assessing antipsychotics in the ICU have several limitations as pointed out above. The scarcity of data calls for well-designed and powered clinical trials. While we wait for those, and in the absence of other effective pharmacological options for the treatment of delirium in the ICU, it is our opinion that antipsychotics can be judiciously used in ICU patients with delirium, particularly in those with agitation.

The data on haloperidol as a prophylactic agent against delirium in the elderly admitted to the ICU after surgery appears promising. However, more studies are needed before haloperidol can be used routinely as a prophylactic agent in this patient population.

## Conclusions

Delirium is common in ICU patients but often goes undetected. Different instruments have been designed to help in the identification of patients with delirium. Whether the implementation of these instruments leads to better outcomes is not fully established. Nonpharmacological approaches, such as physical and occupational therapy, decrease the duration of delirium and should be encouraged. Pharmacological treatment for delirium traditionally includes haloperidol. Second-generation antipsychotics have emerged as an alternative for the treatment of delirium, and they may have a better safety profile. However, to date the studies evaluating these medications have been limited by small sample size. More powered clinical trials are needed to establish the first-line pharmacological treatment for delirium.

## Abbreviations

ICU: Intensive Care Unit; CAM-ICU: Confusion Assessment Method for the Intensive Care Unit.

## Competing interest

The authors have no conflict of interest nor any real or perceived financial interest in any product mentioned in this paper.

## Authors’ contributions

All three authors contributed to writing this manuscript and have reviewed and approved the final version for publication.
